# The Colonoscopy Satisfaction and Safety Questionnaire (CSSQP) for Colorectal Cancer Screening: A Development and Validation Study

**DOI:** 10.3390/ijerph16030392

**Published:** 2019-01-30

**Authors:** Alicia Brotons, Mercedes Guilabert, Francisco Javier Lacueva, José Joaquín Mira, Blanca Lumbreras, María Dolores Picó, Julián Vitaller, Mariana Fe García-Sepulcre, Germán Belda, Javier Sola-Vera

**Affiliations:** 1 Department of Gastroenterology, Vega Baja Hospital of Orihuela, 03314 Orihuela, Spain; germanbelda@gmail.com; 2 Department of Pathology and Surgery, Miguel Hernandez University of Elche, 03203 Elche, Spain; fj.lacueva@umh.es; 3 Department of Health Psychology, Miguel Hernandez University of Elche, 03550 Elche, Spain; mguilabert@umh.es (M.G.); jose.mira@umh.es (J.J.M.); 4 Alicante-Sant Joan Health District, 03550 Alicante, Spain; 5 Department of Public Health, Miguel Hernandez University of Elche, 03550 Elche, Spain; blumbreras@umh.es (B.L.); julian.vitaller@umh.es (J.V.); 6 CIBER of Epidemiology and Public Health, 28029 Madrid, Spain; 7 Department of Gastroenterology, University General Hospital of Elche, 03203 Elche, Spain; madopisa@hotmail.com (M.D.P.); marifegarciasepulcre@gmail.com (M.F.G.-S.); solavera_jav@gva.es (J.S.-V.)

**Keywords:** colonoscopy, patient satisfaction, patient safety, patient experience, colorectal cancer screening program, questionnaire, CSSQP

## Abstract

Colonoscopy services working in colorectal cancer screening programs must perform periodic controls to improve the quality based on patients’ experiences. However, there are no validated instruments in this setting that include the two core dimensions for optimal care: satisfaction and safety. The aim of this study was to design and validate a specific questionnaire for patients undergoing screening colonoscopy after a positive fecal occult blood test, the Colonoscopy Satisfaction and Safety Questionnaire based on patients’ experience (CSSQP). The design included a review of available evidence and used focus groups to identify the relevant dimensions to produce the instrument (content validity). Face validity was analyzed involving 15 patients. Reliability and construct and empirical validity were calculated. Validation involved patients from the colorectal cancer screening program at two referral hospitals in Spain. The CSSQP version 1 consisted of 15 items. The principal components analysis of the satisfaction items isolated three factors with saturation of elements above 0.52 and with high internal consistency and split-half readability: Information, Care, and Service and Facilities features. The analysis of the safety items isolated two factors with element saturations above 0.58: Information Gaps and Safety Incidents. The CSSQP is a new valid and reliable tool for measuring patient’ experiences, including satisfaction and safety perception, after a colorectal cancer screening colonoscopy.

## 1. Introduction

Colorectal cancer is one of the most common cancers in both men and women worldwide [1] and is a good candidate for screening programs. Colonoscopy is an essential procedure in colorectal cancer (CRC) screening programs since its findings have an impact on patient treatment and on the subsequent follow-ups [2,3]. Colonoscopy in the setting of CRC programs is somehow different from conventional colonoscopy. Thus, several studies have shown that patients who undergo CRC screening have higher levels of anxiety, especially those in which the colonoscopy is performed not as a primary test, but after a positive fecal occult blood test (FOBT) [4,5,6].

Endoscopy units working in CRC screening must follow proper controls to improve the quality of colonoscopy procedures. These should be based on patients’ experiences and on health care professionals’ assessments [7]. Patient experience is an important consideration for measuring optimal care [8,9]. In the setting of a CRC screening, it is essential that the patient has a satisfactory colonoscopy experience, as this will improve the adherence of other patients to CRC screenings through word-of-mouth communication [10]. In addition, colonoscopy may be perceived to be a painful and embarrassing procedure and this perception hampers patient participation in screening programs. Therefore, patient experience should be self-reported by patients using a validated scale [11].

While the viewpoint of providers of care has been commonly applied to assess the outcomes of colonoscopy, the patient’s viewpoint has not been systematically included [7].

Several studies have been carried out to measure patient satisfaction after colonoscopy [12,13,14,15,16]; however, most of them have been carried out only from the health professionals’ perspective [17,18,19,20,21,22]. In addition, some studies focused on the assessment of colonoscopy have emphasized the differences in perceptions between patients and health professionals [23].

Although there is some patient-reported experience measures for individuals undergoing colonoscopy, none include the patient’s perception of safety [24,25,26,27]. Therefore, the purpose of this study was to design and validate a specific measure of patient experience, including patient satisfaction and perception of safety, for colonoscopy in the CRC screening program.

## 2. Materials and Methods

### 2.1. Study Design

**Figure 1:**
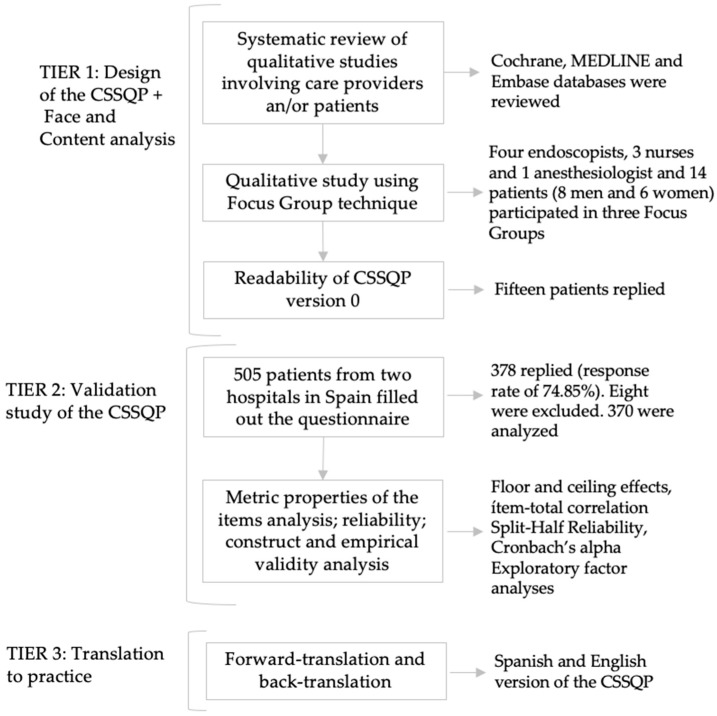
Flow diagram of the Colonoscopy Satisfaction and Safety Questionnaire based on patients’ experience (CSSQP) study.

The design and validation of the Colonoscopy Satisfaction and Safety Questionnaire (CSSQP) considered the face and content validity of this new instrument, as well as the metric properties of their items, reliability, and the construct and empirical validity of this measure [28,29,30]. Finally, the Spanish version was adapted to English using the forward-back-translation procedure (Figure 1).

A systematic literature review was carried out to identify factors associated with positive experiences and perception of safety. Additionally, three focus groups involving physicians, nurses, and patients were applied to explore the dimensions of quality and safety relevant for patients. Finally, areas to be explored and elements for the CSSQP were proposed, which were subsequently validated in patients who underwent a colonoscopy in the colorectal cancer screening program of the National Health Service (NHS).

#### 2.1.1. Factors Contributing to Positive Experiences and Safety Perception

We performed a literature review in the following databases: MEDLINE, Cochrane, and Embase. A combination of terms was applied: “patient satisfaction”; “safety”; “patient-reported outcomes”, “patient experiences”, “colonoscopy”; “endoscopy”, AND “colorectal cancer screening”. The authors reviewed the titles and abstracts of the publications, and they selected pertinent studies [17,23,31,32]. All studies that used qualitative research methodology for the detection of the indicators derived from digestive endoscopy patients were included. Scales validated and standardized by the endoscopy units were considered, such as the modified Group Health Association of America (mGHAA-9) survey and the endoscopy Global Rating Scale (GRS) developed in United Kingdom [14,33]. A meta-narrative review about methods of assessing patient experience in gastrointestinal endoscopy was also used to define the scope of this instrument [12]. Based on this information, key questions and clusters to explore the patients’ experiences after colonoscopy were developed.

#### 2.1.2. Providers’ and Patients’ Views

Qualified staff conducted three group sessions involving providers and patients using key questions and clusters identified. This approach was consistent with the recommendation to identify patients’ perspectives and views thought qualitative research [10,12,34].

The first group consisted of eight health professionals with expertise in CRC screening colonoscopy (four endoscopists, three nurses, and one anesthesiologist). In the other two focus groups, one at each hospital, a random selection of 14 patients participated (eight men and six women, with an age range of 50 to 69 years), including individuals who had undergone a colonoscopy as a part of CRC screening program in the previous 30 days. All participants recruited (endoscopy staff and patients) were informed about the aim of the study and individuals were invited to participate in a focus group session. The sessions continued until no new information emerged and saturation of information was reached.

Content analysis of the data collected during group sessions was performed. The ideas presented by the participants were reorganized into strengths and weaknesses from the patients’ viewpoint. An analysis of the consistency (intra-group and among groups) of those ideas was also carried out. Qualitative data were graded (using a 1—low value—to 5—highest value—scale) considering means and variation coefficients (CV). A CV value less than 0.5 was considered to represent an adequate level of consensus. Results yielded relevant themes from the patients’ experiences.

#### 2.1.3. Reactive Items and Face Validity

Factors related with patients’ experiences were grouped into dimensions, and 28 reactive items were developed to explore them. The wording of the items considered the vocabulary used by the patients involved in the group sessions. Version 0 of the CSSQP included a total of 15 items related to satisfaction, 10 items to safety, and three items used as external control variables. The understanding of these questions was evaluated by 15 patients who had recently followed a screening colonoscopy. Their opinions were also considered in regard to whether these items explored relevant questions (face validity).

### 2.2. Validation Study Subjects

The target population of the validation study included patients who had consecutively undergone a colonoscopy within the CRC screening program between April 2016 and April 2017 at two hospitals: Elche University Hospital and Vega Baja Hospital. These are two referral hospitals for all individuals living in their catchment areas belonging to the NHS in the Autonomous Region of Valencia (Spain). The colorectal cancer screening program of the NHS consists of performing a biennial fecal immunochemical test to people aged 50–69 years. Patients who have a positive FOBT are offered a colonoscopy.

To calculate the sample size, different expected proportions of patients satisfied were calculated for a precision error of 3%, with the most negative at 50, a moderate value at 70, and an optimistic value at 80. The necessary *N* was estimated in each case, ranging from 245 to 384, with an intermediate value of 323. All cases, without losses of responses, used a confidence interval of 95%.

Patients were invited to participate by the endoscopy staff (physician or nurse) who explained the study before being discharged from the endoscopy unit. In both hospitals, colonoscopy was carried out under deep sedation using propofol by the endoscopist; for this reason, patients were given the instruction to fill out the questionnaire at home the day after the colonoscopy procedure, once the effect of the sedation had worn off completely. All participants received an envelope with the necessary materials (invitation letter, anonymous questionnaire, postage-paid envelope) to complete later at home. They were given the instruction to return the questionnaires to a mailbox located in their health center or endoscopy unit.

To avoid missing data, patients received a telephone reminder to fill in the questionnaire between 24 and 72 hours after the colonoscopy. The incomplete questionnaires (less than 50% completed) were classified as “not valid” and were excluded from the statistical analysis.

### 2.3. Data Analysis

#### 2.3.1. Metric Proprieties of the Items

The floor and ceiling effects of each item were analyzed individually in order to assess variability in the gathered data. The “floor” and “ceiling” effect refers to the fact that most participants respond to the same item option (in the upper part or in the lower part of the scale), which means that there is no variability in the answers and, therefore, that item is not useful.

The item-total correlation was also analyzed to characterize the metric proprieties of the elements, excluding those items with low correlations. A minimum of 0.35 Pearson’s correlation was considered acceptable.

#### 2.3.2. Reliability

Cronbach’s alpha was used to estimated internal consistency. Split-half reliability was also estimated using the Spearmen-Brown coefficient since satisfaction escalates over time and test–retest measures could be biased. Items were randomly sorted and then split into two parts before applying this statistic. Kendall coefficient of concordance, assessing agreement/disagreement among raters, was used in the case of dichotomous items (safety scale). A value greater than 0.70 was considered acceptable for all statistics.

#### 2.3.3. Construct Validity

To analyze the construct validity, an exploratory factor analysis to determine the factorial structure of this instrument was conducted using the principal components technique, followed by varimax rotation. Eigenvalues greater than 0.40 and factor loading greater than 0.5 were considered to represent an acceptable level of missing data.

Multivariate analysis was conducted to explain the relationships between sociodemographic variables and CSSQP scores. The odds ratio and its 95% confidence intervals were reported in the logistic regression results. The significance level was set at 5% whenever applicable.

#### 2.3.4. Empirical Validity

Linear regression was used to estimate the predictive capacity of the CSSQP scores. The variables “wait time”, “overall satisfaction”, and “occurrence of complications” were used as external control variables for this analysis. These variables were assessed considering the following items: *“the wait time from when I was told that the fecal blood test was positive until the day of the colonoscopy”, “satisfaction with treatment and the services given during the examination”,* and *“I experienced a complication during or after the colonoscopy”.* Patients who answered the overall satisfaction question as “normal”, “regular”, or “poor” were considered “not satisfied”. Statistical analyses were performed using SPSS software (ver. 20.0, SPSS Inc., Chicago, IL, USA).

### 2.4. Translation-Back Translation

The CSSQP version 1, which consisted of 15 items (Satisfaction scale, 13 items; Perceived safety scale, two items), was initially used in the Spanish version and then was translated into English by a native English speaker with experience in the clinical context. This English version of the CSSQP was translated into Spanish by an independent professional. Both versions were compared by two investigators to solve potential inconsistencies. Supplementary Materials include both versions of this instrument [35].

### 2.5. Ethical Consideration

The study was approved by the ethical committees of the participating hospitals (project identification code PI24/2015) and was conducted in accordance with the Declaration of Helsinki. All participants gave their consent to participate in the study. All the candidates were informed that their decision to participate or not in the study would not affect the standard of care.

## 3. Results

### 3.1. Design of the CSSQP

#### 3.1.1. Literature Review

Relevant dimensions of care to colonoscopy mentioned in previous qualitative studies involving patients were structured into three categories: Information, Care, and Service and Facilities features (see Table 1). The safety perceived by the patient during the colonoscopy procedure was not previously studied and therefore no elements were included in the initial table.

#### 3.1.2. Focus Groups

The main ideas obtained from the focus groups with the participation of health professionals and patients are shown in Table 2. Quality indicators obtained from the patient focus groups were grouped into: Information, Care, Service and Facilities features, and Perceived safety.

Concerning Information indicators, patients highlighted the need for complete information about preparations before the colonoscopy, information provided by the endoscopist about the results of the test, and information regarding telephone numbers and contact channels in case of complications. As for Care indicators, respectful treatment on the part of physicians and nurses throughout the process was highlighted. Participants positively valued privacy and the absence of overcrowding in the room while awaiting the examination. In terms of Service and Facilities features indicators, patients highlighted the waiting time since the date of prescription of the colonoscopy, the ease of the process, its flexibility, the appointment confirmation call, the comfort of the waiting room and recovery area, delays in the waiting room, and the availability of lockers to store personal items during the colonoscopy. Finally, regarding indicators related to safety perception, patients gave priority to the need for clear and accurate information on colonoscopy and sedation risks, as well as what to do in case of complications.

### 3.2. Validation of the CSSPQ

#### 3.2.1. Sample Description

During the study period, a total of 505 consecutive patients underwent colonoscopy after a FOBT, 300 (59.4%) at Vega Baja Hospital and 205 (40.6%) at Elche University Hospital. Three hundred and seventy-eight 378 filled out the questionnaire (global response rate 74.85%, 80% at Vega Baja Hospital of Orihuela and 63% at University Hospital of Elche). Eight questionnaires were incomplete and interpreted as “not valid”, and therefore were excluded. The demographic characteristics of the 370 patients with a valid questionnaire are presented in Table 3.

A total of 39 safety incidents were reported by 34 patients (9.2% of patients with a valid questionnaire). There were no serious safety incidents, such as perforation or bleeding, that required intervention or admission to the hospital.

Answers to the question about overall satisfaction showed that 79.5% of the patients were “satisfied” (36.5% very good and 43.0% excellent) versus 20.5% who were “not satisfied” (19.7% normal and 0.8% regular).

#### 3.2.2. Items Analysis, Reliability, and Validity

Floor and ceiling effects were not identified to eliminate any of the elements in either the satisfaction or the perceived safety questionnaire. Two items were excluded due to low item-total correlation (<0.5): “*the care they took when asking me if I had done the preparation correctly, what medication was I taking or if I had any allergy to foods or medications”;* and *“management of pain (abdominal pain, flatulence, nausea, etc.) as a result of the colonoscopy”.*

Cronbach’s alpha was 0.86 and the Spearman-Brown coefficient was 0.85, suggesting adequate reliability. For the analysis of the validity of CSSQP version 0, in the satisfaction scale (Table 4), the analysis of the principal components revealed three factors that explained 64% of the variance, with element saturation over 0.52: Information, which includes five items; Care, with five items; and Service and Facilities features, with three items. Isolated factors showed adequate predictive capacity considering external control variables. The average scores of each of the factors were as follows: Information 3.7 (*SD* 0.7); Care, 4.1 (*SD* 0.6); Service and Facilities features, 2.9 (*SD* 0.8).

Concerning the safety scale, the analysis of the principal components (Table 5) revealed two factors with element saturation over 0.58: Information Gaps and Safety Incidents. Kendall coefficient of concordance assessing agreement among raters was 0.71 for the safety scale, reflecting coherent differences between patients suffering safety incidents and patient without this experience. Both factors were related to the occurrence of complications during colonoscopy. The average scores of each factor were as follows: Information Gaps, 1.2 (*SD* 0.2) and Safety Incidents, 2.0 (*SD* 0.1). Since the frequency of safety incidents was low, the final version of the perceived safety items was clustered to reduce the number of questions in the questionnaire, using those with higher occurrence.

#### 3.2.3. Relationship between Items’ Scores and Sociodemographic Variables

All the items generally showed scores defined as good. The worst rated question was related to the changing area and the space to store the personal belongings: *Changing area, wardrobe, and lockers.*

Significant differences were observed in the indicators derived from the groups (Information, Care, and Service and Facilities features) according to sex and studies. For example, men considered that information on the preparation prior to colonoscopy was clearer and more useful than women (OR 1.71; 95% CI 1.09–2.68, *p* = 0.020). Also, they considered that discomfort after colonoscopy had been handled better (OR 1.83; 95% CI 1.17–2.85, *p* = 0.008). Moreover, patients with secondary and university studies considered that the information on the preparation prior to colonoscopy was clearer and more useful than patients with primary studies (OR 3.13; 95% CI 1.51–6.51, *p* = 0.002 and OR 5.04; 95% CI 1.81–14.09, *p* = 0.002, respectively); patients with secondary and university studies also considered that prior information on indications and risks of the procedure was clearer and more useful than patients with primary studies (OR 2.48; 95% CI 1.17–5.22, *p* = 0.017 and OR 3.47; 95% CI 1.31–9.19, *p* = 0.012, respectively). No differences were observed in the indicators derived from the groups according to the following variables: center, age, marital status, and first colonoscopy.

Overall satisfaction was not related to sex, education, marital status, or experience of previous colonoscopy. Five items of the CSSQP were the best predictive factors of overall satisfaction: *Treatment and behavior received from the endoscopy staff* (OR 2.65, 95% CI 1.47–4.80), *Treatment and behavior of the doctor who carried out the procedure* (OR 2.41, 95% CI 1.30–4.48), *Useful information on the day of the colonoscopy prior to the procedure* (OR 2.35, 95% CI 1.33–4.14), *Changing area, wardrobe, and lockers (safety and comfort)* (OR 1.66, 95% CI 1.06–2.61), and *Efficiency of the methods to reduce pain used during the colonoscopy* (OR 1.57, 95% CI 1.03–2.39).

#### 3.2.4. Additional Comments from the Patients

With respect to the additional comments, patients’ principal observations included: adaptation of waiting and dressing rooms, more comfortable recovery and waiting rooms, more privacy in recovery room, and shorter waiting time in the waiting room. These observations were considered in the development of the final questionnaire.

#### 3.2.5. CSSQP Version 1

The final version of the CSSQP included three sections: (a) a satisfaction scale, with 13 items; (b) a perceived safety scale, with two items; and (c) a space to include additional comments.

The satisfaction scale was based on a rating scale with a numerical range of 1 to 5: poor (1), regular (2), good (3), very good (4), and excellent (5). The perceived safety scale was composed of three items with dichotomous response options: yes (1) or no (2) (Supplementary Materials). The CSSQP score was calculated only when the patients had responded to at least 50% of the questions. This version was translated into English by a native English speaker not related to the study with experience in English-Spanish-English translation of health reports and papers (forward-translations and back-translations procedure).

## 4. Discussion

We have designed and validated an instrument to assess the satisfaction and perceived safety of patients undergoing a colonoscopy procedure after positive FOBT (as a part of a CRC screening program) using both qualitative and quantitative methods. The questionnaire aims to explore all the areas that fall within the scope of the colonoscopy services working in screening programs.

Our subject of study was the patients who underwent a colonoscopy after a positive FOBT within the colon cancer screening program. These patients have peculiarities with respect to patients who attend a colonoscopy for other reasons (evaluation of symptoms, surveillance of polyps, or even colonoscopy as primary screening test). Several studies have shown that these patients have high levels of anxiety [4,5,6]. Therefore, the expectations of these patients about the colonoscopy can differ substantially from the rest of patients, therefore it is essential for health professionals to have knowledge of these expectations.

Our study provides, for the first time, a colonoscopy questionnaire in the setting of CRC screening program taking into consideration patients’ opinions through a qualitative phase with focus groups. Also, it should be noted that the most innovative aspect of our questionnaire is the incorporation of safety items derived from patients’ experiences, which had never been taken into account before. The instrument is presented in both Spanish and English to facilitate its use.

During the focus group phase, patients made similar observations about the colonoscopy process to those made in other studies [16,17,18,23,36]. However, our patients were not worried about pain or discomfort during colonoscopy, in contrast to other studies [37,38]. It should be considered that in our study all the colonoscopies were carried out under deep sedation. In this context, patients did not feel pain during colonoscopy, only slight discomfort in the recovery room.

Health professionals’ perspectives were also included in the present study, and we observed differences compared to the patients’ perspectives, as reported in other studies [23]. Health professionals took more seriously the care during the colonoscopy (pain and discomfort) and they highlighted the discomfort caused by the preparation prior to colonoscopy. However, in the focus groups with patients we observed that both the information and care were pointed out as the most important aspects of the process. These results suggest that patients must be involved in the design of questionnaires evaluating colonoscopy units.

The response rate of patients in the validation of the questionnaire was higher than in other studies [23] (global response rate of 74.8%), which is probably due to the high level of acceptance of the screening program, the high level of acceptance of this instrument, and the telephone reminder.

Overall, a positive experience was observed, with a high level of satisfaction and safety perception. However, women evaluated the items of Information received and Care to be worse than men, similar to previous reports [39,40,41]. According to our results, we have changed some clinical practices in our endoscopy units, such as reduction of the waiting time in the waiting room before colonoscopy. We have also improved the privacy in the recovering area, with toilets separated by gender and lockable lockers.

Several questionnaires have been developed to evaluate patient satisfaction after an endoscopic procedure, but they present significant differences with respect to ours. Thus, the mGHAA-9 survey [14], developed entirely from the perspective of health professionals, did not include quality indicators that our study found to be relevant for patients, such as information on the preparation process, the perception of risks, the necessity of the colonoscopy, and the efficiency of anesthesia for pain reduction. The GRS [33] identified 12 areas considered important for patients who undergo an endoscopy, but only one measure of patient comfort during endoscopy was formally validated.

The most relevant study related to colonoscopy in the CRC screening program was carried out in Great Britain [37] using questionnaires developed exclusively from the perspective of health professionals. Another study based on patient-reported measures was carried out with patients from the Polish screening program. This study identified several independent, modifiable factors associated with pain during and after colonoscopy [42]. None of the studies previously mentioned considered safety indicators from the patient perspective. In our questionnaire, we have inserted perceived safety as an outcome related to the patient experience. This measure has not been included in other scales before.

This study has some limitations. One of them is that only two Spanish hospitals participated in the study. It must be kept in mind that there is certain heterogeneity among hospitals in terms of how the CRC screening program works and therefore these results might not apply to every center. Also, although the response rate was high, it cannot be discarded that non-responders were less satisfied. A limitation to empirical validity is that the questionnaires were totally anonymous, so it was not possible to collect data from the non-responders. Test–retest measures (reproducibility) were not calculated because satisfaction level increases with time since the test is performed, especially when patients undergo sedation. Although in our study construct validity was calculated through exploratory factor analysis, CSSQP requires additional studies to explore convergent/discriminant validity comparing this instrument with other tools to measure patients’ satisfaction. Lastly, the study was done in patients who underwent colonoscopy after a positive FOBT, so the results may not be applicable to participants who attend colonoscopy as a primary screening test. For this reason, external validation studies with different CCR screening programs are needed.

The leading gastrointestinal endoscopy societies now recommended that patient satisfaction should be routinely collected as one of the core quality indicators. For this purpose, the CSSQP could be used routinely in CRC screening programs.

## 5. Conclusions

This study presents a validated questionnaire for colonoscopy as a part of CRC screening developed from patient experiences and not only the perspective of health professionals. Our results identify new indicators for quality and safety in regard to information and healthcare considerations based on patient-reported outcomes.

Including the CSSQP as a standardized procedure could be useful in order to know the patient’s perception of different areas and to introduce a new measure related to safety. These indicators collected on a regular basis could be used to implement programs for continuous improvement in colonoscopy services.

## Figures and Tables

**Table 1:** Guide to determine aspects of importance for patients who have undergone a colonoscopy.

INFORMATION	CARE	SERVICE/FACILITIES FEATURES
Clear instructions and detailed information about the process and preparation	Treated with consideration and respect	Aesthetics aspects of the waiting room, changing areas, wardrobe, restroom, work room, recovery areas
Previous warning and reminders for the colonoscopy appointment	Proper control of pain and complications	Presence of health professionals during the colonoscopy
Information related to “what to do” in case of a complication	Adequate sedation	Waiting time between the fecal blood test and the colonoscopy procedure
Waiting time for notification of results	Individual treatment	Flexibility of schedules for the colonoscopy appointment
Access to diagnostic follow-up and feedback mechanisms related to the service	Confidence derived from proper treatment and professionalism	Flexibility to choose: endoscopist, gender of those present in the room, sedation
Contact phone numbers and/or accessibility of schedule information	Privacy	Civility of the endoscopy staff
		Process safety (colonoscopy procedure, physical environment, and medical staff)

**Table 2:** Results of the focus group with patients and health professionals.

PATIENTS	Patients		PROFESSIONALS	Professionals	
	Mean	CV		Mean	CV
Complete information for preparation before colonoscopy	5.0	0.0	Complete information for preparation before colonoscopy	4.8	0.0
Clarity of information before colonoscopy	5.0	0.0	Proper and friendly treatment from the attending health personnel	4.8	0.0
Telephone appointment reminder prior to the colonoscopy	5.0	0.0	Colonoscopy carried out without pain and discomfort (during colonoscopy)	4.8	0.0
Trust in waking up peacefully	5.0	0.0	Discomfort and pain after colonoscopy	4.67	0.1
Experienced professionals (they know what they are doing)	4.9	0.1	Waiting time between the notification of a positive fecal blood test (FOBT) and the colonoscopy procedure	4.3	0.2
Complete information for preparation before colonoscopy	5.0	0.0	Punctuality of the colonoscopy procedure process (without a prior wait)	4.1	0.2
Waiting time between the notification of a positive fecal blood test (FOBT) and the colonoscopy procedure	4.7	0.1	Trust in waking up peacefully	3.8	0.1
Punctuality of the colonoscopy procedure process (without a prior wait)	4.6	0.2	Displeasure in preparation for the colonoscopy (type of diet to be followed, control of diabetic patients, control of anticoagulants and antiplatelet drugs)	3.3	0.3
Good performance of the procedure (from arrival to departure)	4.6	0.2	Specific diet before colonoscopy (liquid diet)	2.83	0.3
Information from the endoscopist carrying out the colonoscopy prior to the patients return home related to how the procedure went (polyps, “all is well”, etc.)	4.6	0.1			
Trust in professionals	4.6	0.2			
Proper informed consent on behalf of the nurse on the day of the patient’s arrival for the colonoscopy	4.5	0.2			
Prior information is in line with actual development	4.4	0.1			
Reasonable recovery time (between 30 and 60 minutes) in the box after the colonoscopy	4.4	0.2			
Recovery room conditions (comfortable)	4.3	0.3			
Introduction to the endoscopist before testing	4.3	0.3			
Contact telephone (questions, in case of something going wrong…)	4.3	0.3			
Lack of privacy in the waiting room	4.3	0.3			
Proper equipment	4.3	0.3			
Information regarding medication/allergies	4.3	0.3			
Information prior to the procedure regarding what sensations to expect after the colonoscopy	4.1	0.2			
Need for a place (locker) to store personal objects during the procedure	3.7	0.5			

The scale ranges from 1 to 5, with 5 being the highest score. CV: coefficient of variance.

**Table 3:** Demographic characteristics of the 370 patients with a valid questionnaire.

Variable	*N*	%
**Centre**		
Elche	130	35.1
Orihuela	240	64.9
**Sex**		
Men	227	61.4
Women	143	38.6
**Age (mean, *SD*)**	60.7 ± 5.2	
**Education**		
No studies	51	13.8
Primary studies	189	51.1
Secondary studies	96	25.9
University studies	32	8.6
NA	2	0.5
**Marital status**		
Single	18	4.9
Married	286	77.3
Separated	37	10.0
Widowed	24	6.5
Other	5	1.4
**First colonoscopy**		
Yes	316	85.4
No	53	14.3
NA	1	0.3
**Years since last colonoscopy (mean, *SD*)**	8.7 ± 7.5	

*SD*: standard deviation; NA: Not Available. Percentages may not add up to exactly 100 per cent, owing to rounding off.

**Table 4:** Selected questions from the satisfaction scale in the CSSQP version 0 and internal consistency of the components.

Question Number	Matrix of Rotated Questions	Information 1	Care 2	Service Environment and Facilities 3
**14**	Useful information after the colonoscopy	0.82		
**13**	Information from the physician related to results and opportunity to ask questions	0.79		
**9**	Useful information on the day of the colonoscopy, prior to the procedure	0.73		
**2**	Prior information on indications and risks of the colonoscopy	0.69		
**1**	Useful and clear information on the preparation, prior to the colonoscopy	0.51		
**4**	Waiting time on the day of the colonoscopy (waiting room)		0.72	
**15**	Treatment and behavior on behalf of the endoscopy personnel (nurses and assistants)		0.67	
**6**	Communication ability of endoscopy personnel to resolve worries and doubts		0.64	
**10**	Effective anesthesia during the colonoscopy (pain and discomfort)		0.61	
**16**	Treatment and behavior of the endoscopist carrying out the procedure		0.60	
**7**	Comfortable and secure bathrooms and dressing rooms			0.83
**12**	Comfort of the recovery room			0.73
**5**	Comfort of the waiting area			0.70
	**Explained Variance**	44.2%	10.9%	8.0%
	**Predictive/Empirical Validity (Waiting time)**	B = 0.39 (0.23–0.56), *p* = 0.000	B = 0.24 (0.06–0.43), *p* = 0.011	B = 0.08 (−0.04–0.21), *p* = 0.195
	**Predictive/Empirical Validity (Overall Satisfaction)**	B = 0.30 (0.18–0.41), *p* = 0.000	B = 0.52 (0.40–0.65), *p* = 0.000	B = 0.08 (−0.01–0.17), *p* = 0.054

Extraction method: Analysis of principal components; Rotation method: Kaiser normalization with varimax.

**Table 5:** Selected questions from the safety scale in the CSSQP version 0 and internal consistency of the components.

Question Number	Matrix of Rotated Components	Reported Gaps in the Information Received	Reported Safety Incidents
1	Necessary and sufficient information prior to the colonoscopy	0.77	
3	Proper indications and instructions that were similar among health professionals	0.76	
4	Information about anesthesia	0.58	
7	Allergic reactions to medication or health materials used		0.75
8	Accidents during the colonoscopy		0.74
9	Confusion with documentation or identity on the day of the colonoscopy		0.66
	**Explained Variance**	24.6%	27.6%
	**Predictive/Empirical Validity (occurrence of complications)**	B = −0.17 (−0.25, −0.09), *p* < 0.0001	B = 0.32 (0.14–0.50), *p* < 0.0001

Extraction method: Analysis of principal components; Rotation method: Kaiser normalization with varimax.

## Supplementary Materials

The following are available online http://www.mdpi.com/1660-4601/16/3/392/s1.
