# Supplementary material for: The Colonoscopy Satisfaction and Safety Questionnaire (CSSQP) for Colorectal Cancer Screening: A Development and Validation Study
Source: Int J Environ Res Public Health. 2019 Jan 30;16(3):392. doi: 10.3390/ijerph16030392 (PMC6388170; doi:10.3390/ijerph16030392)
Supplement: Supplementary file 1 [file ijerph-16-00392-s001.pdf]

ANNEX I: CSSQP version 1

COLONOSCOPY SATISFACTION AND SAFETY QUESTIONNAIRE BASED ON PATIENT EXPERIENCE (CSSQP)

INSTRUCTIONS

- This questionnaire aims to assess your colonoscopy experience in order to improve the endoscopy services.
- There are not right or wrong answers, we are only interested in your opinion.
- This questionnaire is voluntary and anonymous.
- Thank you for helping us to improve by providing your responses.

PERSONAL INFORMATION:

A) Age: ☐

B) Gender:

(1) Female ☐ (2) Male ☐

C) Level of education:

(1) No education ☐ (2) Primary education ☐
(3) Secondary education (high school, professional) ☐ (4) University studies ☐

D) Marital status:

(1) Single ☐ (2) Married ☐ (3) Separated ☐ (4) Widowed ☐
(5) Other ☐

E) Is this your first colonoscopy? (1) Yes ☐ (2) No ☐

If you answered No, how many years have passed since your last colonoscopy? ☐

	Mark with an “X” the response that best reflects your level of satisfaction	POOR	REGULAR	GOOD	VERY GOOD	EXCELLENT
1	**Clarity and usefulness** of the information received in preparation to the colonoscopy					
2	The explanations about the **risks of the colonoscopy procedure** and the reasons why it was being recommended					
3	**Time spent in the endoscopy waiting room** until I was called to undergo the procedure					
4	**Comfort of the waiting room** in the endoscopy unit					
5	**Attitude of endoscopy staff** in answering my questions and worries before the colonoscopy					
6	**Changing area, wardrobe and lockers** (safety and comfort)					
7	Information I was given the day of the colonoscopy about **what I should do and what I could feel during the colonoscopy** (sensations, possible discomfort, etc.)					
8	Efficiency of the **methods to reduce pain used** during the colonoscopy					
9	**Comfort and privacy of the recovery room** where I stayed after the procedure					
10	**Information received from the doctor just after finishing the procedure** related to the results					
11	Information after the colonoscopy about **how I would feel once at home** and **what** I **would need to do** in case of any emergency (contact telephone to call if needed)					
12	**Treatment and behaviour** received from the endoscopy staff (nurses and assistants) the day of the procedure					
13	**Treatment and behaviour** of the doctor who carried out the procedure					
A	**In general, the satisfaction with the treatment and services provided during the procedure were…**					
B	**Waiting time since I was informed of the positive faecal blood test until the colonoscopy took place**					

	Mark with an "X" the response that best reflects your feeling about the level of safety	YES	NO
1	I received **sufficient information** about the colonoscopy before the procedure (preparation, possible complications, interactions with medicines, anaesthesia, proper indications and instructions that were similar among health professionals, etc.)		
2	There was an incident on the day of the colonoscopy (confusion related to my **documentation or identity, allergic reaction to the medication or materials used, trauma, fracture or hematoma, etc.)**		
A	**I experienced a complication during or after the colonoscopy (intake, operation, emergency room, etc.)**		

COMMENTS AND SUGGESTIONS (Make any observation or suggestion that you would like to add related to the quality and safety of the colonoscopy procedure carried out):

THANK YOU FOR TAKING YOUR TIME TO FILL THE QUESTIONNAIRE

CUESTIONARIO DE SATISFACCIÓN Y SEGURIDAD PERCIBIDA POR LOS PACIENTES QUE SE HAN REALIZADO UNA COLONOSCOPIA DE CRIBADO DE CÁNCER COLORRECTAL (CSSQP)

INSTRUCCIONES

- con la intención de mejorar nuestra práctica clínica habitual.
- No hay respuestas correctas o incorrectas, sólo nos interesa su opinión.
- Responder a este cuestionario es totalmente voluntario y anónimo.
- Gracias por ayudarnos a mejorar facilitándonos sus respuestas.

DATOS PERSONALES:

F) Edad: ☐

G) Sexo:

(1) Mujer ☐ (2) Hombre ☐

H) Nivel de estudios:

(1) Sin estudios ☐ (2) Estudios Primarios ☐
(3) Estudios Secundarios (Bachiller, FP) ☐ (4) Estudios Universitarios ☐

I) Estado civil:

(1) Soltero/a ☐ (2) Casado/a ☐ (3) Separado/a ☐ (4) Viudo/a ☐
(5) Otros ☐

J) ¿Es la primera vez que se hace una colonoscopia? (1) SÍ ☐ (2) NO ☐

Si la respuesta es NO, indique cuántos años hace de la última colonoscopia ☐

	Marque con una X la respuesta que mejor refleje su experiencia con la colonoscopia	MALA (1)	REGULAR (2)	BUENA (3)	MUY BUENA (4)	EXCELENTE (5)
1	La **claridad y utilidad de la información** recibida para prepararme antes de la colonoscopia					
2	Las explicaciones sobre los **riesgos de la colonoscopia y por qué me aconsejaban** que me hiciera esta prueba					
3	El **tiempo que pasé en la sala de espera** del Servicio de Endoscopia hasta que me llamaron para realizarme la prueba					
4	La **comodidad de la sala de espera** del Servicio de Endoscopia					
5	La **actitud** del personal del Servicio de Endoscopia para solucionar mis dudas y preocupaciones antes de la colonoscopia					
6	El **espacio** que había para cambiarme de ropa y para guardar mis objetos personales (seguridad y comodidad)					
7	La información que me dieron el día de la colonoscopia sobre **qué debía hacer yo y qué podía ir experimentando** (sensaciones, posibles molestias, etc.) para que no me preocupara					
8	La eficacia de la anestesia para **reducir el dolor** durante la colonoscopia					
9	La **comodidad y privacidad de la sala de recuperación** donde esperé tras la colonoscopia					
10	La **información que recibí del médico justo después de acabar la colonoscopia** sobre los resultados					
11	La información que me dieron después de la colonoscopia sobre **cómo me iba a sentir en casa** (gases, molestia, etc.) y cómo tenía que actuar si tenía alguna urgencia (teléfono de contacto para llamar en caso de necesidad)					
12	El **trato y el comportamiento** del personal de endoscopia (enfermería y auxiliares) que recibí el día de la colonoscopia					
13	El **trato y comportamiento** del médico que realizó la prueba					
A	**En general, la satisfacción en el trato y los servicios prestados durante la colonoscopia han sido…**					
B	**El tiempo de espera desde que me solicitaron la prueba hasta el día que me realizaron la colonoscopia**					

	Marque con una X la respuesta que mejor refleje su sensación de seguridad respecto a la colonoscopia	SÍ (1)	NO (2)
1	Recibí **suficiente información** sobre la colonoscopia antes de la realización (preparación, posibles complicaciones, interacciones con mis medicamentos, anestesia, indicaciones e instrucciones correctas y similares de todos los profesionales sanitarios, etc.)		
2	Hubo algún **incidente** el día de la colonoscopia (confusión en mi documentación o identidad, reacción alérgica por la medicación o material utilizado, traumatismo, fractura o hematoma, etc.)		
A	Sufrí alguna complicación durante o después de la colonoscopia (sangrado anal, ingreso, operación, acudir a urgencias, etc.)		

COMENTARIOS ADICIONALES (Escriba cualquier observación o sugerencia que desee añadir relacionada con la calidad y seguridad de la colonoscopia realizada):

MUCHAS GRACIAS POR PARTICIPAR EN ESTE ESTUDIO Y CONTESTAR EL CUESTIONARIO
